# Investigating the Genetics of Hippocampal Volume in Older Adults without Dementia

**DOI:** 10.1371/journal.pone.0116920

**Published:** 2015-01-27

**Authors:** Karen A. Mather, Nicola J. Armstrong, Wei Wen, John B. Kwok, Amelia A. Assareh, Anbupalam Thalamuthu, Simone Reppermund, Konsta Duesing, Margaret J. Wright, David Ames, Julian N. Trollor, Henry Brodaty, Peter R. Schofield, Perminder S. Sachdev

**Affiliations:** 1 Centre for Healthy Brain Ageing, School of Psychiatry, University of New South Wales (UNSW), Sydney, Australia; 2 School of Mathematics and Statistics, University of Sydney, Sydney, Australia; 3 School of Mathematics and Statistics, UNSW, Sydney, Australia; 4 Neuroscience Research Australia, Sydney, Australia; 5 School of Medical Sciences, UNSW, Sydney, Australia; 6 The Commonwealth Scientific and Industrial Research Organisation (CSIRO) Animal Food and Health Sciences Division, Sydney, Australia; 7 QIMR Berghofer Medical Research Institute, Brisbane, Australia; 8 National Ageing Research Institute, Royal Melbourne Hospital, Melbourne, Australia; 9 Academic Unit for Psychiatry of Old Age, University of Melbourne, Melbourne, Australia; 10 Department of Developmental Disability Neuropsychiatry, UNSW, Sydney, Australia; 11 Primary Dementia Collaborative Research Centre, UNSW, Sydney, Australia; 12 Neuropsychiatric Institute, Prince of Wales Hospital, Sydney, Australia; Johns Hopkins University, UNITED STATES

## Abstract

Hippocampal atrophy is observed with ageing and age-related neurodegenerative disease. Identification of the genetic correlates of hippocampal volume (HV) and atrophy may assist in elucidating the mechanisms of ageing and age-related neurodegeneration. Using two community cohorts of older Caucasians we estimated the heritability of HV and examined associations of HV with previously identified single nucleotide polymorphisms (SNPs). In addition we undertook genome-association studies (GWAS) examining HV and HV atrophy. Participants were community-dwelling non-demented older adults from the longitudinal Sydney Memory and Ageing Study (Sydney MAS) (*N* = 498) and the Older Australian Twins Study (OATS) (*N* = 351) aged 65 and over. HV was measured using T1-weighted magnetic resonance images. Heritability of HV was estimated in OATS. Genome-wide genotyping was imputed using the 1K Genomes reference set. Associations with HV-candidate and Alzheimer’s disease (AD)-related SNPs were investigated. A GWAS examining HV (in both cohorts) and an exploratory GWAS of HV atrophy over two years (in Sydney MAS only) were also undertaken. HV heritability was estimated at 62–65%. The previously identified GWAS HV SNP (rs6581612) and the candidate *BDNF* SNP (rs6265) were nominally significant (*p* = 0.047 and *p* = 0.041 respectively). No AD-related SNPs, including the *APOE* ε4 polymorphism, were significant. No significant results were observed for either of the GWAS undertaken. Despite our estimate of a high heritability of HV, our results are consistent with a highly polygenic model suggesting that SNPs identified from prior studies, including GWAS meta-analyses, can be difficult to replicate in smaller samples of older adults.

## INTRODUCTION

The hippocampus is implicated in memory and learning, and hippocampal atrophy is associated with neuropsychiatric and neurodegenerative disorders, including Alzheimer’s disease (AD) [1]. Hippocampal volume (HV) also declines with normal ageing [2], yet our understanding of the causal factors leading to hippocampal atrophy is rudimentary. As episodic memory performance also declines with ageing [3, 4] and the hippocampus is a key structure for memory, it is vital to gain a better understanding of the molecular processes underlying the age-related decline in HV volume. Since the heritability of HV is moderate to high in adults (40–74%) [1, 5, 6], genetic studies may prove fruitful for identification of genetic polymorphisms associated with HV, thereby elucidating the underlying biology.

Candidate gene studies have identified several polymorphisms associated with HV, but the results have been variable, with the most consistent results observed for *APOE* ε4 carriers and lower HV [7, 8]. In addition, a recent meta-analysis found that Met allele carriers of the *BDNF* Val66Met polymorphism (rs6265) had lower HV than non-carriers [9].

These inconsistent findings with candidate genes have led to collaborations being formed to undertake genome-wide association studies (GWAS) examining HV in large cohorts. The CHARGE consortium [10], using a sample of over 9,000 non-demented older adults (mean age = 67 years), found several HV-associated SNPs that were both genome-wide significant in the discovery sample and which replicated in a meta-analysis of independent replication cohorts. The ENIGMA consortium [1] observed only one SNP (rs7294919) that was genome-wide significant in a younger cohort of 7,795 individuals (mean age = 40 years), which included individuals with psychiatric disorders including AD. Moreover, the effect of rs7294919 remained significant when only healthy individuals were considered (*N* = 5775). Only rs7294919 was found to be significant in both the CHARGE and ENIGMA cohorts. The discrepancy in results may be due to differences in the age of the cohorts and the different covariates employed. A third GWAS by Melville et al. [11], investigated a sample comprising 2,102 older adults, (overall mean = 73.2years), which also included individuals with AD, mild cognitive impairment and controls. The authors observed several results, which did not overlap with either CHARGE or ENIGMA’s genome-wide significant results.

In this study, we used two community cohorts of non-demented older Caucasians to investigate the genetics of HV and atrophy. As few studies have reported the heritability of HV in older samples of both men and women we estimated HV heritability in our relatively large sample to be moderate to high. We also sought to replicate the published results of CHARGE, ENIGMA and Melville et al, as well as investigating known candidate genes for HV. However, only two SNPs showed any evidence of replication. In addition, we conducted our own GWAS in an attempt to identify novel SNPs associated with both HV and HV atrophy in older Caucasians and found no genome-wide significant results.

## MATERIALS AND METHODS

### Ethics Statement

The Sydney Memory and Ageing Study was approved by the Human Research Ethics Committees of the University of New South Wales and the Illawarra Area Health Service. The Older Australians Twins Study was approved by the appropriate ethics committees of the Australian Twin Registry, University of New South Wales, University of Melbourne, Queensland Institute of Medical Research and the South Eastern Sydney and Illawarra Area Health Service. All participants provided written informed consent and the data was de-identified and anonymised before use.

### Samples

**Sydney Memory and Ageing Study (Sydney MAS)**. Participants were recruited randomly from the compulsory electoral roll of two Sydney (Australia) suburbs and were aged 70–90 years. Exclusion criteria included a dementia diagnosis, limited English or a medical/psychological condition that would prevent them from completing assessments, an age and education-adjusted MMSE score of <24, psychotic symptoms or a diagnosis of schizophrenia/bipolar disorder, multiple sclerosis, motor neuron disease, developmental disability and/or progressive malignancy. Participants are followed-up every two years with a repeat assessment. Of the 1,037 participants at baseline, the mean age was 78.84 years and 44.8% were men. Full details of the sample are given elsewhere [12]. A subsample was used in the present study that had both HV and genome-wide genotyping data available. As shown in Table 1, for the HV volume cross-sectional analyses there were 498 participants (278 males, 220 females). For HV atrophy analyses, there were 325 participants with HV information available for both Waves of data collection and genetic data (Table 1).

**Table 1:** Descriptive statistics for Sydney MAS and OATS participants with hippocampal volume and genotyping data available.

	**MAS Wave 1^a^**	**MAS Wave 2^b^**	**OATS^a^**
Sample size (*N*)	498	325	351
Age, *M* (SD)	78.45 (4.70)	79.83 (4.46)	70.42 (5.06)
Number females (%)	220 (44.18)	173 (53.23)	231 (65.8)
Total HV (mm^3^), *M* (SD)	6746.07 (837.18)	6554.784 (899.70)	7186.72 (824.65)
Mean Biannual HV Atrophy %^c^ (SD)	NA	-3.49 (5.69)	NA

**Notes**. MAS = Sydney Memory & Ageing Study; OATS = Older Australian Twins Study; HV = hippocampal volume;

^a^Data presented for those with both HV & genetic data;

^b^Data presented for those with Wave 1 & 2 HV & genetic data;

^c^(Wave 2 -Wave1/Wave 1) *100;

NA = not available

**Older Australian Twins Study (OATS)**. Participants who were aged 65 years and over were recruited from the Australian Twin Registry and through a recruitment drive. Inclusion criteria included an ability to consent, a co-twin who consented to participate, completion of some education in English and at least a low average IQ (≥80), as estimated from the National Adult Reading Test (NART, Nelson & Willison, 1991), a reading test for estimating pre-morbid intellectual functioning. Exclusion criteria included inadequate English to complete the assessment and/or a current diagnosis of an acute psychosis. At baseline there were 623 participants with a mean age of 70.77 years and 34.8% were men. For further details see Sachdev et al. [13]. A subsample was used for this study, which was comprised of individuals with both HV and genetic data available. Any dementia cases were excluded. As shown in Table 1, the final sample consisted of 120 males and 231 females (*N* = 351). HV data was available for Wave 1 only.

### Hippocampal Volume (HV)

Participants of both the Sydney MAS and OATS studies were invited to undergo a brain magnetic resonance imaging (MRI) scan.

**Sydney MAS MRI Acquisition**. After excluding participants due to contraindications (e.g. pacemaker) 542 individuals underwent a MRI brain scan. Approximately half were scanned using a Philips 3T Intera Quasar scanner (Philips Medical Systems, Netherlands) whilst the remaining participants were scanned on a Philips 3T Achieva Quasar Dual scanner. At the second Wave all brain scans (*N* = 421) were undertaken using the Achieva Quasar Dual scanner. For further details see Jiang et al. [14].

**OATS MRI Acquisition**. After excluding any participants due to contraindications, 414 participants underwent a brain MRI scan. Since participants were recruited across a number of different research centres, MRI data were obtained on four different scanners. Twin pairs were always scanned on the same scanner and were scanned either on the same day or temporally very close to each other (<few weeks). For further details see Batouli et al. [15].

**HV/intracranial volume (ICV) calculation**. In both studies, 3D T1-weighted scans were processed with the FMRIB Software Library (FSL) v5.2.0 [16–18]. Visual quality control of HV results were performed using ENIGMA protocols (http://enigma.loni.ucla.edu/protocols/imaging-protocols/protocol-for-quality-control-and-summary-statistics/). Scans were excluded if they failed visual quality control. Intracranial volume (ICV) was calculated from the sum of grey matter, white matter and cerebrospinal fluid. Hippocampal volume was estimated as described in Jiang et al. for Sydney MAS [14] and Batouli et al. for OATS [15].

For MAS participants with both Wave 1 and Wave 2 MRI scans, the FMRIB’s Linear Image Registration Tool (FLIRT, v5.5) [19] was used to linearly register the 2nd brain scan of each participant onto their 1st scan. FMRIB’s Integrated Registration and Segmentation Tool (FIRST v4.1) [20] was then used to generate hippocampal volumes according to the fitted shape model. For further details see Jiang et al., [14]. Atrophy was defined as decline in average bilateral HV over 2 years (Wave 1-Wave 2).

### Genotyping and Imputation

**Genome-wide Genotyping**. *Sydney MAS:* There were 972 DNA samples available. Genome-wide genotyping was undertaken using the Affymetrix Genome-wide Human SNP Array 6.0 (California, USA) at the Ramaciotti Centre, University of New South Wales, Sydney, Australia, following the recommended protocol. Six samples were not successfully genotyped due to either insufficient DNA concentration or failed genotyping.

The CRLMM package (v1.10.0) in R (v2.12.1) was used to call genotypes. Affymetrix Release 31 SNP 6 annotations were used (31/01/11). There were 966 participants and 905,028 SNP markers. SNPs were excluded if the genotyping call rate was <95%, had a minor allele frequency <0.01 or if they failed a Hardy-Weinberg equilibrium threshold of <1 × 10^-6^. Participants were excluded from analysis if the call rate was <95% (*N* = 1). Identity-by-descent analysis in PLINK [21] determined that there were three pairs of related individuals (siblings) and one of each pair was excluded from further analyses. Population stratification was assessed using EIGENSTRAT [22] and 17 significant principal components (PCs) were extracted. A number of ethnic outliers were identified and excluded (*N* = 37). In addition, multi-dimensional scaling (MDS) components were obtained using PLINK. After final QC checks there were 925 Sydney MAS participants (417 men, 508 women) with data for 734,550 SNPs with a mean genotyping call rate of 99.5%. Imputation was undertaken to the 1K Genomes reference set (phase 1 release v3) using MACH/minimac according to the ENIGMA2 protocol (see http://enigma.loni.ucla.edu/wp-content/uploads/2012/07/ENIGMA21KGP_cookbook_v3.doc). SNPs with poor imputation quality were omitted from any further analyses (*R^2^*≤0.6).

*OATS:* DNA samples (*N* = 548) were genotyped at the University of Queensland Diamantina Institute, Brisbane, Australia, using the Illumina Omni Express array (California, USA) following the manufacturer’s instructions and 733,202 genotypes were called using Illumina Genome Studio V2011.1. Quality control procedures were the same as used for Sydney MAS. No individuals were removed due to low genotyping rates. Family relationships were assessed using identity by descent in PLINK and sex checks were undertaken, which resulted in 6 samples being excluded due to unexpected relatedness or incorrect sex. EIGENSTRAT analysis identified 25 ethnic outliers that were removed from further analyses. Three significant principal components were found. In addition, multi-dimensional scaling (MDS) components were obtained using PLINK. After QC, there was information on 646,791 SNPs for 517 individuals (182 male, 335 females) with a mean genotyping call rate of 99.9%. Imputation to the 1K Genomes reference set was undertaken using MACH/minimac and followed the ENIGMA2 protocol (see above).

**APOE ε2/3/4 Polymorphism**. Genotyping was performed using Taqman assays (Applied Biosystems Inc., Foster City, CA, USA) for the two SNPs rs7412 and rs429358 as described in Song et al. [23]. The two SNPs were in Hardy-Weinberg equilibrium. Participants were classified into two categories, either carriers of the ε4 allele or not.

### Selection of HV-Related SNPs for Replication

A total of 28 SNPs were selected: Six genome-wide significant SNPs were extracted from the results of three recent GWAS [1, 10, 11]. For the CHARGE [10] and ENIGMA [1] studies, SNPs were selected if they were considered genome-wide significant and showed evidence of replication. For Melville et al., [11] SNPs were selected if genome-wide significant for the meta-analysis of the entire sample. The top ten ranked genetic polymorphisms for late-onset AD were retrieved from the Alzgene online database (http://alzgene.org accessed 10/12/12) and used in the analyses. HV-relevant SNPs (*N* = 12) identified by Stein et al., [1] were also investigated.

### Heritability

Estimates of heritability were calculated for average bilateral HV using 87 monozygotic and 65 dizygotic pairs (92 female, 36 male same-sex pairs and 24 opposite sex pairs). Analyses were performed using structural equation modelling as implemented in OpenMx [24] adjusting for age, sex and scanner +/- ICV. Firstly, the full model including additive genetic variance (A), shared environment (C) and the unique environment components (E) (ACE model) was estimated. To identify the most parsimonious model, AE, CE and E models were then compared with the ACE model.

### Statistical Analyses

**Replication Analyses**. For CHARGE consortium identified HV SNPs, the covariates were age and sex [10]. For ENIGMA HV SNPs, the covariates were age, sex, age^2^, age × sex interaction, age^2^ × sex interaction, 4 MDS, dummy variables for the different scanners and +/- ICV [1]. For the Melville et al. [11] study SNPs, the covariates were age, sex and ICV. For the AD-related SNPs, the covariates were age and sex to enable comparison with the CHARGE consortium that undertook a similar analysis. For the other SNP analyses, the appropriate covariates were used according to prior analyses. A *p*-value of ≤.05 was considered significant.

**GWAS of Sydney MAS and OATS**. To increase power, we undertook a meta-analysis of the GWAS results from Sydney MAS and OATS. For each individual cohort, linear regression analyses were run using average bilateral HV and the appropriate covariates using Mach2qtl [25, 26] for MAS and merlin [27] for the entire OATS sample, together with the imputed dosage data. Model 1 was adjusted for age, sex and scanner. Model 2 was additionally adjusted for ICV. An inverse-variance weighted meta-analysis of Sydney MAS and OATS GWAS results was performed using METAL software [28]. For the longitudinal HV atrophy GWAS in Sydney MAS, analyses were run as above but used genotyped data only (*N* = 734,550 SNPs) in PLINK. Results were considered significant if *p*<5×10^-8^ and suggestive if *p*<5×10^-5^ for genome-wide analyses.

Power calculations suggested that we had 70% or higher power based on the sample size (n = 849), if the effect size is at least 115 and the allele frequency is greater than 25% at a nominal 5% level of significance using Quanto (see http://hydra.usc.edu/gxe).

## RESULTS

As shown in Table 1, the Sydney MAS cohort was older and larger than OATS. The OATS study had more females. As expected, total HV was greater in the younger OATS sample. For Sydney MAS, which was the only cohort to have longitudinal data available, average total HV decreased over two years at a mean rate of ~3.5%. HV atrophy was not correlated with HV at baseline (*r* = 0.013, *p* = 0.820).

### Heritability

Heritability for HV was estimated in the OATS sample using structural equation modelling. The parsimony of the models with genetic, shared and unique environmental components was examined by comparing the likelihoods of the full ACE model versus the reduced models AE (p = 1), CE (p = 4.014e-4), E (p = 8.002e-12). Since the AE model resulted in an identical log likelihood to the ACE model, this model was selected as the most parsimonious and used to estimate heritability. Heritability for HV when adjusted for age, sex and scanner was 0.65 (95% C.I. = 0.51, 0.75). Inclusion of intracranial volume (ICV) as an additional covariate yielded a heritability of 0.62 (95% C.I. = 0.48, 0.73).

### Replication of Prior Genome-wide Significant Results for HV

As shown in Table 2, only one of the 6 SNPs examined, rs6581612 (identified by CHARGE [10]), was nominally significant in the meta-analysis (*p* = 0.047). The observed effect was in the same direction as the original results. This result was most likely driven by the result for the Sydney MAS cohort (*p* = 0.044). No SNP was associated with HV in the OATS sample. The genome-wide significant SNP identified by both CHARGE and ENIGMA, rs729419, was not significantly associated with HV, irrespective of the covariates used. SNPs that were identified by Melville et al. [11] as genome-wide significant did not replicate in our cohorts.

**Table 2:** Replication results of prior genome-wide significant HV SNPs for Sydney MAS and OATS by cohort and meta-analysis.

**SNP**	**Chr**	**Covariates**	**Effect Allele**	**MAS *B (SE) N* = 498**	**MAS *p*-value**	**OATS *B (SE) N* = 351**	**OATS *p*-value**	**MAS/ OATS meta-analysis *B (SE)***	**MAS/ OATS meta-analysis *p*-value**	**GWAS Reference**
rs17178006	12	age, sex	G	-23.924 (48.039)	0.619	-77.133 (60.153)	0.200	-44.64 (37.54)	0.234	(CHARGE)[10]
rs6581612	12	age, sex	C	-58.785 (29.159)	0.044*	-24.201 (39.720)	0.542	-46.67 (23.50)	0.047*	(CHARGE)[10]
rs7294919	12	age, sex, age^2^, age x sex, age^2^ x sex, 4 MDS	C	-35.552 (37.743)	0.346	-84.832 (49.937)	0.080	-39.68 (30.98)	0.200	(ENIGMA) [1]
rs7294919	12	age, sex	C	-38.707 (37.915)	0.307	-48.202 (52.229)	0.374	-41.82 (31.07)	0.178	(CHARGE) [10]
rs7294919	12	age, sex, age^2^, age x sex, age^2^ x sex, 4 MDS, ICV	C	-26.753 (35.870)	0.456	-65.344 (46.935)	0.164	-26.56 (29.32)	0.365	(ENIGMA) [1]
rs6703865	1	age, sex, ICV	A	43.511 (42.085)	0.301	-43.917 (61.651)	0.476	15.72 (35.76)	0.651	[11]
rs2298948	2	age, sex, ICV	C	-28.684 (24.991)	0.251	-31.721 (31.633)	0.316	-29.85 (19.61)	0.128	[11]
rs9315702	13	age, sex, ICV	A	30.053 (23.780)	0.206	12.438 (31.091)	0.689	23.55 (18.89)	0.212	[11]

***Notes***. MDS = multi-dimensional components; ICV = intracranial volume; Sydney MAS = Sydney Memory & Ageing Study, OATS = Older Australian Twins Study;

* *p*-value≤.05

### Late-onset Alzheimer’s disease Candidate SNPs

No evidence of association with HV was observed for the 10 top-ranked genetic variants for late-onset AD in either the Sydney MAS/OATS cohorts or in the meta-analysis (Table 3).

**Table 3:** HV Association results examining the top 10 ranked genetic polymorphisms for late-onset Alzheimer’s Disease in the CHARGE discovery meta-analysis, Sydney MAS, OATS and meta-analyses of MAS/OATS.

**Gene**	**Variant**	**Chr**	**CHARGE *p*-value^a, b^*N* ≥6946**	**MAS *p*-value *N* = 498^b^**	**OATS *p*-value *N* = 351^b^**	**Meta-analysis MAS/OATS *p*-value**	**Effect allele meta-analysis**	**Direction of effect in meta-analysis**
*APOE^c^*	*APOE* ε4	19	Did not examine directly	0.754	0.476	NA	NA	NA
*BIN1*	rs744373	2	0.03*	0.221	0.726	0.540	A	-+
*CLU*	rs11136000	8	0.8	0.265	0.673	0.519	T	+-
*ABCA7*	rs3764650	19	0.3	n.a.	0.475	n.a.	NA	NA
*CR1*	rs3818361	1	0.09	0.288	0.439	0.643	A	-+
*PICALM*	rs3851179	11	1.0	0.117	0.940	0.230	T	+-
*MS4A6A*	rs610932	11	0.5	0.310	0.593	0.651	T	-+
*CD33*	rs3865444	19	NA	0.678	0.544	0.485	A	++
*MS4A4E*	rs670139	11	0.001**	0.380	0.670	0.659	T	+-
*CD2AP*	rs9349407	6	NA	0.054	0.124	0.466	C	-+

**Notes**. Alzgene results from http://alzgene.org, accessed 10/12/12;

^a^From [10];

^b^ covariates: age & sex;

^c^ *APOE ε4* carriers vs non-carriers;

Sydney MAS = Sydney Memory & Ageing Study; OATS = Older Australian Twins Study; n.a.: Not applicable due to poor imputation quality for this SNP; NA: not available;

* *p*≤.05;

** *p*≤.001

### Candidate Genetic Variants for Hippocampal Volume

There were no significant results for HV-relevant genetic variants in either of the two cohorts (Table 4). In the meta-analysis, however, rs6265 (*BNDF*), was nominally significant (*p* = 0.041).

**Table 4:** HV association of genetic variants previously identified as having relevance to HV (Stein et al. 2012) in ENIGMA, Sydney MAS, OATS and a meta-analysis of MAS/OATS.

**Gene**	**SNP (proxy)**	**Chr**	**ENIGMA *p*-value^a, b^*N* = 5776**	**MAS *p*-value^b^*N* = 498**	**OATS *p*-value^b^*N* = 351**	**Meta-analysis MAS/OATS**	**Effect allele meta-analysis**	**Direction of effect in meta-analysis**
*DISC1*	rs821616	1	NA	0.384	0.829	0.445	A	++
*DISC1*	(rs1754606; *R^2^* = 1.0)	1	0.372	0.318	0.804	0.395	T	++
*DTNBP1*	rs1011313	6	0.849	0.306	0.827	0.685	T	+-
*DTNBP1*	rs1018381	6	NA	0.499	0.230	0.918	A	+-
*DTNBP1*	(rs875463; *R^2^* = 1.0)	6	0.767	0.576	0.229	0.975	A	+-
*NRG1*	rs35753505	8	NA	0.113	0.560	0.076	T	--
*NRG1*	(rs12681411 *R* ^2^ = 0.835)	8	0.499	0.094	0.501	0.060	C	--
*CLU*	rs11136000	8	0.636	0.447	0.869	0.783	T	+-
*BDNF*	rs6265	11	0.887	0.090	0.214	0.041*	T	--
*PICALM*	rs3851179	11	0.030*	0.251	0.909	0.280	T	--
*TOMM40*	rs2075650	19	0.663	n.a.	0.956	n.a.	NA	NA
*COMT*	rs4680	22	0.200	0.930	0.117	0.272	A	+-

**Notes**. In the ENIGMA analyses, a proxy (in high LD) was used if the named SNP was not available;

^a^ From [1];

^**b**^ ENIGMA covariates: age, sex, age^2^, age × sex interaction, age^2^ × sex interaction, 4 multi-dimensional components (MDS), dummy variables for different scanners, ICV (intracranial volume);

Sydney MAS = Sydney Memory & Ageing Study; OATS = Older Australian Twins Study; n.a.: Not applicable due to poor imputation quality for this SNP; NA: not available;

* *p*≤.05

### HV GWAS Meta-Analysis

A GWAS for HV was undertaken in the two cohorts using cross-sectional data. The genomic inflation factor was approximately 1.1 for Sydney MAS and 1.0 for OATS. There were no significant results; however, a number of suggestive results were observed (S1 and S2 Tables, S1 and S2 Fig.). None of the observed suggestive GWAS results overlapped with the significant/suggestive results reported by CHARGE [10], ENIGMA [1] or by Melville et al [11].

### Exploratory GWAS for Hippocampal Atrophy

A GWAS for HV atrophy over 2 years was undertaken in the Sydney MAS cohort. There were no genome-wide significant results for either model (age, sex, +/- ICV, S3–S4 Tables). The top results were similar between the two models but did not overlap with the results from our baseline cross-sectional GWAS. Of those HV-relevant SNPs identified from previous studies and AD-risk SNPs (Tables 2–4) that were available in the genotyped sample (16/28 SNPs), none were significantly associated with HV atrophy, including the *BDNF* SNP, rs6265.

## DISCUSSION

In this study we investigated the genetics of HV in two samples of community-dwelling older adults. Our results confirm prior evidence that HV in older adults has moderate to high heritability with 62–65% of HV variance being explained by genetic factors. We also undertook candidate gene analyses, including examining results from three prior GWAS, and found nominally significant results for a candidate *BDNF* SNP (rs6265) and a previous GWAS result (rs6581612). A meta-analysis of genome-wide analysis results examining HV in our two modest sized cohorts of older adults did not find any significant results. Furthermore, as HC atrophy is one of the earliest markers of dementia, we undertook an exploratory GWAS examining hippocampal atrophy over 2 years with no genome-wide significant results observed. To our knowledge, this is one of the first longitudinal studies to examine the genetics of hippocampal atrophy using a GWAS.

Our results for heritability (*h^2^*) of HV in older adults are comparable to findings for young Australian adult twins (20–30 yrs, *h^2^* = 0.62), Mexican-Americans (18–85 yrs, *h^2^* = 0.74) [1] and Dutch young adults (range of means 21.3–32.0 yrs, *h^2^* for L HV = 0.73, *h^2^* for R HV = 0.78) [29]. Our heritability results are higher than that reported by Sullivan et al.[6] (*h^2^* = 0.40) using a similarly aged sample (68–78 yrs). However, they utilised a smaller sample (*N* = 84 pairs) and examined men only. Together, these results suggest that HV is heritable over the lifespan.

In this study, replication of previously GWAS-identified SNPs associated with HV was undertaken. We were unable to replicate the SNP, rs7294919, which both the CHARGE and ENIGMA consortia reported as genome-wide significant, despite using different covariates and samples. However, one previously identified GWAS HV SNP, (CHARGE consortium, [10]), rs6581612, showed nominal significance in our meta-analysis. This SNP is located near the genes *WIF1* and *LEMD3* on chromosome 12. The *WIF1* gene product, Wnt inhibitory factor 1, is a secreted protein that inhibits the activity of extracellular WNT proteins. Inhibition of WNT proteins has been implicated in the degeneration of mature mouse hippocampal neurons [30].

We investigated previously identified SNPs relevant to hippocampal structure from the literature prior to the recent GWAS [1]. In our cohorts, only the previously reported *BDNF* SNP, rs6265, showed nominal evidence of replication. The effect of the *BDNF* SNP was in a similar direction to that observed by a recent meta-analysis where the Met allele was significantly associated with lower hippocampal volume [9]. Although there is some evidence that *APOE* ε4 carriers have smaller HV (e.g. R HV only in young adults) [8], this was not observed in our study. However, our results are supported by other studies in older adults. Jak et al. [31], in a sample of adults aged 63–92 years found that ε4 carrier status was not associated with cross-sectional HV. Moreover, a more recent study found no relationship between HV and *APOE* ε4 carrier status in a sample of over 400 older adults [32]. The ENIGMA consortium found only one significant SNP in their analysis (*PICALM*), which was not significant in the CHARGE analyses or our samples. Overall, these results suggest that previous results from genetic studies may suffer from the winner’s curse phenomenon and/or that it is difficult to replicate results across studies using different samples and measures.

Hippocampal atrophy is an early marker of AD. Therefore, we investigated the top SNPs previously associated with AD. CHARGE had reported the results for several of these SNPs and observed two SNPs that were associated with HV in their large sample, which were located in the *BIN1* and *MS4A6A* genes [10]. However, none of these SNPs were associated with HV in our study. Our results and those of CHARGE suggest that many of the top ranked AD-related SNPs have minimal or no effect on HV in the early stages of the disease and/or that the effect is masked in samples that contain individuals who may and may not develop AD.

Our discovery GWAS meta-analysis of the Sydney MAS and OATS cohorts revealed no genome-wide significant results for HV. This most likely reflects the relatively small size of our cohorts compared to recently published studies, such as Bis et al. [10]. In an extension of this work, we examined the genetics of hippocampal atrophy, a putative endophenotype for AD and found no genome-wide significant results. However, longitudinal data were only available for Sydney MAS. Further, hippocampal atrophy may be more influenced by environmental processes and disease such as amyloidopathy, which may decrease the likelihood of identifying genetic factors that predispose an individual to increased risk of atrophy. In the future, heritability estimates will assist in defining the genetic contribution to HV atrophy. Larger studies and/or HV longitudinal data collected over a longer period of time may be required to better examine the genetics of HV atrophy.

A limitation of this study is that we did not correct for multiple-testing and the nominally significant results in our replication analyses would not survive such a correction. Further, our sample sizes were smaller than previous studies. The use of multiple scanners within and across cohorts is also an additional limitation. However, this is a common problem in similar studies and was adjusted for in our GWAS analyses. Furthermore, in regards to our GWAS, there are differences in sample size and age between the previously reported GWAS and our cohorts. Our samples were substantially smaller than the CHARGE and ENIGMA cohorts and older than the ENIGMA cohort. The Melville et al. sample was of similar age but included individuals with mild cognitive impairment and AD in addition to cognitively normal individuals, aiming to enrich the sample for hippocampal atrophy. Our samples were community-based and those with dementia were excluded. Thus our sample was searching for genetic correlates of HV and HV atrophy in relatively healthy older individuals.

In conclusion, we examined the genetics of hippocampal volume and atrophy by investigating candidate gene variants in non-demented community-dwelling older Australians. In *a priori* analyses, one previously identified SNP, rs6581612, and a *BDNF* variant showed nominal significant HV associations in our cohorts. No genome-wide significant results were observed in our discovery GWAS for hippocampal atrophy or in our HV GWAS. Due to the relatively small size of our cohorts, further larger studies are warranted to determine whether prior results for HV, including the recent GWAS consortia results, can be replicated in other community samples. Future studies should aim to determine whether different genetic variants can be identified across the lifespan. Longitudinal analyses over a longer period (>2 years) may also prove more productive for identifying genetic variants associated with hippocampal atrophy. Moreover, despite significant heritability, it appears that consistent with prior genetic studies of complex traits, many genes of small effect influence HV and atrophy.

## Supporting Information

S1 Table Cross-sectional hippocampal volume GWAS meta-analysis top results when using age, sex and scanner type as covariates. (DOCX)

S2 Table Cross-sectional hippocampal volume GWAS meta-analysis top results when using age, sex, scanner type and ICV as covariates. (DOCX)

S3 Table Top GWAS results for hippocampal atrophy over two years for Sydney MAS participants with age and sex as covariates. (DOCX)

S4 Table Top GWAS results for hippocampal atrophy over two years for Sydney MAS participants with age, sex and ICV as covariates. (DOCX)

S1 Fig Genome-wide Manhattan plot for hippocampal volume using the Sydney Memory and Ageing Study Cohort. P-values for each individual SNP are plotted against chromosome position from the results of the association analysis with bilateral hippocampal volume. The analysis was adjusted for age, sex, scanner and ICV.
(DOCX)

S2 Fig Genome-wide Manhattan plot for hippocampal volume using the Older Australian Twins Cohort. P-values for each individual SNP are plotted against chromosome position from the results of the association analysis with bilateral hippocampal volume. The analysis was adjusted for age, sex, scanner and ICV.
(DOCX)

## References

[1] Stein JL, Medland SE, Vasquez AA, Hibar DP, Senstad RE, et al (2012) Identification of common variants associated with human hippocampal and intracranial volumes. Nat Genet 44: 552–561. 10.1038/ng.2250 22504417 PMC3635491

[2] Fox NC, Schott JM (2004) Imaging cerebral atrophy: normal ageing to Alzheimer′s disease. Lancet 363: 392–394. 10.1016/S0140-6736(04)15441-X 15074306

[3] Hedden T, Gabrieli JD (2004) Insights into the ageing mind: a view from cognitive neuroscience. Nature reviews Neuroscience 5: 87–96. 10.1038/nrn1323 14735112

[4] Morrison JH, Baxter MG (2012) The ageing cortical synapse: hallmarks and implications for cognitive decline. Nature reviews Neuroscience 13: 240–250. 10.1038/nrn3200 22395804 PMC3592200

[5] Blokland GA, de Zubicaray GI, McMahon KL, Wright MJ (2012) Genetic and environmental influences on neuroimaging phenotypes: a meta-analytical perspective on twin imaging studies. Twin Res Hum Genet 15: 351–371. 10.1017/thg.2012.11 22856370 PMC4291185

[6] Sullivan EV, Pfefferbaum A, Swan GE, Carmelli D (2001) Heritability of hippocampal size in elderly twin men: equivalent influence from genes and environment. Hippocampus 11: 754–762. 10.1002/hipo.1091 11811670

[7] Mueller SG, Weiner MW (2009) Selective effect of age, Apo e4, and Alzheimer′s disease on hippocampal subfields. Hippocampus 19: 558–564. 10.1002/hipo.20614 19405132 PMC2802542

[8] O′Dwyer L, Lamberton F, Matura S, Tanner C, Scheibe M, et al (2012) Reduced hippocampal volume in healthy young ApoE4 carriers: an MRI study. PLoS One 7: e48895 10.1371/journal.pone.0048895 23152815 PMC3494711

[9] Molendijk ML, Bus BA, Spinhoven P, Kaimatzoglou A, Oude Voshaar RC, et al (2012) A systematic review and meta-analysis on the association between BDNF val(66)met and hippocampal volume--a genuine effect or a winners curse? American journal of medical genetics Part B, Neuropsychiatric genetics: the official publication of the International Society of Psychiatric Genetics 159B: 731–740. 10.1002/ajmg.b.32078 22815222

[10] Bis JC, DeCarli C, Smith AV, van der Lijn F, Crivello F, et al (2012) Common variants at 12q14 and 12q24 are associated with hippocampal volume. Nat Genet 44: 545–551. 10.1038/ng.2237 22504421 PMC3427729

[11] Melville SA, Buros J, Parrado AR, Vardarajan B, Logue MW, et al (2012) Multiple loci influencing hippocampal degeneration identified by genome scan. Ann Neurol 72: 65–75. 10.1002/ana.23644 22745009 PMC3405172

[12] Sachdev PS, Brodaty H, Reppermund S, Kochan NA, Trollor JN, et al (2010) The Sydney Memory and Ageing Study (MAS): methodology and baseline medical and neuropsychiatric characteristics of an elderly epidemiological non-demented cohort of Australians aged 70–90 years. International psychogeriatrics / IPA 22: 1248–1264. 10.1017/S1041610210001067 20637138

[13] Sachdev PS, Lammel A, Trollor JN, Lee T, Wright MJ, et al (2009) A comprehensive neuropsychiatric study of elderly twins: the Older Australian Twins Study. Twin research and human genetics: the official journal of the International Society for Twin Studies 12: 573–582. 10.1375/twin.12.6.573 19943720

[14] Jiang J, Sachdev P, Lipnicki DM, Zhang H, Liu T, et al (2013) A longitudinal study of brain atrophy over two years in community-dwelling older individuals. NeuroImage. 10.1016/j.neuroimage.2013.08.022 23959201

[15] Batouli SA, Sachdev PS, Wen W, Wright MJ, Ames D, et al (2013) Heritability of brain volumes in older adults: the Older Australian Twins Study. Neurobiology of aging. 10.1016/j.neurobiolaging.2013.10.079 24231518

[16] Smith SM, Jenkinson M, Woolrich MW, Beckmann CF, Behrens TE, et al (2004) Advances in functional and structural MR image analysis and implementation as FSL. NeuroImage 23 Suppl 1: S208–219. 10.1016/j.neuroimage.2004.07.051 15501092

[17] Woolrich MW, Jbabdi S, Patenaude B, Chappell M, Makni S, et al (2009) Bayesian analysis of neuroimaging data in FSL. NeuroImage 45: S173–186. 10.1016/j.neuroimage.2008.10.055 19059349

[18] Jenkinson M, Beckmann CF, Behrens TE, Woolrich MW, Smith SM (2012) Fsl. NeuroImage 62: 782–790. 10.1016/j.neuroimage.2011.09.015 21979382

[19] Jenkinson M, Bannister P, Brady M, Smith S (2002) Improved optimization for the robust and accurate linear registration and motion correction of brain images. NeuroImage 17: 825–841. 10.1006/nimg.2002.1132 12377157

[20] Patenaude B, Smith SM, Kennedy DN, Jenkinson M (2011) A Bayesian model of shape and appearance for subcortical brain segmentation. NeuroImage 56: 907–922. 10.1016/j.neuroimage.2011.02.046 21352927 PMC3417233

[21] Purcell S, Neale B, Todd-Brown K, Thomas L, Ferreira MA, et al (2007) PLINK: a tool set for whole-genome association and population-based linkage analyses. American journal of human genetics 81: 559–575. 10.1086/519795 17701901 PMC1950838

[22] Price AL, Patterson NJ, Plenge RM, Weinblatt ME, Shadick NA, et al (2006) Principal components analysis corrects for stratification in genome-wide association studies. Nature genetics 38: 904–909. 10.1038/ng1847 16862161

[23] Song F, Poljak A, Crawford J, Kochan NA, Wen W, et al (2012) Plasma apolipoprotein levels are associated with cognitive status and decline in a community cohort of older individuals. PloS one 7: e34078 10.1371/journal.pone.0034078 22701550 PMC3372509

[24] Boker, SM, Neale MC, Maes HH, Wilde MJ, et al. (2012) OpenMx 1.3 User Guide.

[25] Li Y, Willer C, Sanna S, Abecasis G (2009) Genotype imputation. Annual review of genomics and human genetics 10: 387–406. 10.1146/annurev.genom.9.081307.164242 19715440 PMC2925172

[26] Li Y, Willer CJ, Ding J, Scheet P, Abecasis GR (2010) MaCH: using sequence and genotype data to estimate haplotypes and unobserved genotypes. Genetic epidemiology 34: 816–834. 10.1002/gepi.20533 21058334 PMC3175618

[27] Abecasis GR, Cherny SS, Cookson WO, Cardon LR (2002) Merlin—rapid analysis of dense genetic maps using sparse gene flow trees. Nature genetics 30: 97–101. 10.1038/ng786 11731797

[28] Willer CJ, Li Y, Abecasis GR (2010) METAL: fast and efficient meta-analysis of genomewide association scans. Bioinformatics 26: 2190–2191. 10.1093/bioinformatics/btq340 20616382 PMC2922887

[29] den Braber A, Bohlken MM, Brouwer RM, van ′t Ent D, Kanai R, et al (2013) Heritability of subcortical brain measures: A perspective for future genome-wide association studies. NeuroImage 83C: 98–102. 10.1016/j.neuroimage.2013.06.027 23770413

[30] Kim H, Won S, Hwang DY, Lee JS, Kim M, et al (2011) Downregulation of Wnt/beta-catenin signaling causes degeneration of hippocampal neurons in vivo. Neurobiology of aging 32: 2316 e2311–2315. 10.1016/j.neurobiolaging.2010.03.013 20409609

[31] Jak AJ, Houston WS, Nagel BJ, Corey-Bloom J, Bondi MW (2007) Differential cross-sectional and longitudinal impact of APOE genotype on hippocampal volumes in nondemented older adults. Dementia and geriatric cognitive disorders 23: 382–389. 10.1159/000101340 17389798 PMC2084479

[32] Ferencz B, Laukka EJ, Lovden M, Kalpouzos G, Keller L, et al (2013) The influence of APOE and TOMM40 polymorphisms on hippocampal volume and episodic memory in old age. Frontiers in human neuroscience 7: 198 10.3389/fnhum.2013.00198 23734114 PMC3660657

