# Supplementary material for: Investigating the Genetics of Hippocampal Volume in Older Adults without Dementia
Source: PLoS One. 2015 Jan 27;10(1):e0116920. doi: 10.1371/journal.pone.0116920 (PMC4308067; doi:10.1371/journal.pone.0116920)
Supplement: S1 Table — (DOCX) [file pone.0116920.s001.docx]

**SUPPLEMENTARY MATERIAL**

**Table S1.** Cross-sectional hippocampal volume GWAS meta-analysis top results when using age, sex and scanner type as covariates

| **SNP** | **CHR** | **BP** | **Effect Allele** | **Beta (SE)** | ***P*-value** | **Effect Direction** | **Gene** | **Feature** |
| --- | --- | --- | --- | --- | --- | --- | --- | --- |
| rs8033195 | 15 | 94872686 | A | 174.53 (34.59) | 4.51E-07 | ++ | *MCTP2* | intron |
| rs950173 | 2 | 172570419 | A | -167.40 (34.10) | 9.12E-07 | -- | *DYNC1I2* | intron |
| rs1019623 | 11 | 127686353 | T | -107.87 (22.34) | 1.38E-06 | -- | - | - |
| rs10790922 | 11 | 127681080 | A | -109.21 (22.72) | 1.53E-06 | -- | - | - |
| rs13000153 | 2 | 172586067 | A | 156.68 (32.65) | 1.60E-06 | ++ | *DYNC1I2* | intron |
| rs1863254 | 11 | 127683994 | T | -105.90 (22.27) | 1.98E-06 | -- | - | - |
| rs890134 | 11 | 127679755 | T | 108.89 (22.99) | 2.18E-06 | ++ | - | - |
| rs35419961 | 9 | 11930364 | A | -366.98 (77.52) | 2.20E-06 | -- | - | - |
| rs10790921 | 11 | 127680050 | T | 107.21 (22.70) | 2.33E-06 | ++ | - | - |
| rs2874910 | 11 | 127683774 | C | -103.84 (22.01) | 2.38E-06 | -- | - | - |
| rs750637 | 11 | 127685302 | A | -103.59 (22.00) | 2.49E-06 | -- | - | - |
| rs1863255 | 11 | 127680217 | T | 109.84 (23.45) | 2.82E-06 | ++ | - | - |
| rs13023239 | 2 | 205761832 | A | -112.73 (24.09) | 2.88E-06 | -- | *PARD3B* | intron |
| rs11221065 | 11 | 127689882 | T | 106.34 (22.74) | 2.93E-06 | ++ | - | - |
| rs7931878 | 11 | 127683476 | T | -106.35 (22.8) | 3.10E-06 | -- | - | - |
| rs7931009 | 11 | 127688725 | T | -106.10 (22.78) | 3.20E-06 | -- | - | - |
| rs6504535 | 17 | 65773808 | T | 118.38 (25.44) | 3.28E-06 | ++ | - | - |
| rs10790923 | 11 | 127688549 | C | 104.56 (22.50) | 3.38E-06 | ++ | - | - |
| rs13031723 | 2 | 205763480 | T | 112.27 (24.19) | 3.48E-06 | ++ | *PARD3B* | intron |
| rs55951657 | 14 | 74074732 | A | -158.61 (34.21) | 3.54E-06 | -- | - | - |
| rs12951929 | 17 | 65774409 | C | -117.64 (25.52) | 4.03E-06 | -- | - | - |
| rs12226793 | 11 | 127694191 | A | -105.58 (22.92) | 4.11E-06 | -- | - | - |
| rs13033719 | 2 | 205758481 | A | -103.27 (22.43) | 4.15E-06 | -- | *PARD3B* | intron |
| rs148621641 | 12 | 78772439 | A | 425.19 (92.48) | 4.27E-06 | ++ | - | - |
| rs857160 | 1 | 57241867 | T | -100.41 (21.85) | 4.32E-06 | -- | *C1orf168* | intron |
| rs1774817 | 1 | 57243086 | T | -100.33 (21.85) | 4.40E-06 | -- | *C1orf168* | intron |
| rs61308264 | 11 | 127693084 | T | 105.23 (22.92) | 4.41E-06 | ++ | - | - |
| rs262147 | 7 | 158745142 | T | 150.06 (32.71) | 4.49E-06 | ++ | - | - |
| rs4791118 | 17 | 65775272 | T | -116.27 (25.38) | 4.61E-06 | -- | - | - |
| rs2630772 | 12 | 54426521 | T | 110.21 (24.09) | 4.75E-06 | ++ | *HOXC6* | 3’ |
| rs139293074 | 5 | 58083195 | A | 1064.6 (233.00) | 4.90E-06 | ++ | *RAB3C* | intron |
| rs142494129 | 12 | 95161353 | T | 1015.12 (222.40) | 5.01E-06 | ++ | - | - |
| rs8073934 | 17 | 65773705 | T | -116.12 (25.49) | 5.23E-06 | -- | - | - |
| rs72763355 | 1 | 238785309 | A | -297.72 (65.38) | 5.28E-06 | -- | - | - |
| rs8068596 | 17 | 65773157 | A | -115.72 (25.43) | 5.33E-06 | -- | - | - |
| rs8018361 | 14 | 74073502 | T | -155.31 (34.17) | 5.48E-06 | -- | - | - |
| rs8019162 | 14 | 74073324 | T | 155.08 (34.15) | 5.60E-06 | ++ | - | - |
| rs6574129 | 14 | 74066812 | T | -155.07 (34.15) | 5.61E-06 | -- | - | - |
| rs9671218 | 14 | 74065505 | A | -155.09 (34.17) | 5.65E-06 | -- | - | - |
| rs2009884 | 14 | 74061490 | T | -156.53 (34.5) | 5.70E-06 | -- | *ACOT4* | intron |
| rs1475622 | 13 | 82716163 | C | -221.18 (48.82) | 5.89E-06 | -- | - | - |
| rs61907470 | 11 | 127696697 | T | 104.96 (23.17) | 5.90E-06 | ++ | - | - |
| rs12101151 | 14 | 74064151 | T | -155.66 (34.38) | 5.95E-06 | -- | *ACOT4* | 3’ |
| rs55689428 | 14 | 74064245 | T | -155.6 (34.36) | 5.96E-06 | -- | *ACOT4* | 3’ |
| rs9672127 | 14 | 74065221 | A | -155.19 (34.3) | 6.06E-06 | -- | - | - |
| rs12587542 | 14 | 74067562 | A | -154.52 (34.16) | 6.10E-06 | -- | - | - |
| rs857161 | 1 | 57240469 | T | 100.29 (22.18) | 6.12E-06 | ++ | *C1orf168* | intron |
| rs7936160 | 11 | 127680781 | A | -101.77 (22.51) | 6.12E-06 | -- | - | - |
| rs7140298 | 14 | 74056564 | T | 160.50 (35.57) | 6.44E-06 | ++ | *ACOT4* | 5’ |
| rs7159970 | 14 | 74056288 | A | 160.54 (35.58) | 6.44E-06 | ++ | *-* | - |
| rs28494539 | 14 | 74054934 | T | -160.66 (35.63) | 6.52E-06 | -- | *-* | - |
| rs12588475 | 14 | 74056820 | A | 159.95 (35.5) | 6.62E-06 | ++ | *ACOT4* | 5’ |
| rs7160971 | 14 | 74056604 | A | -160.04 (35.53) | 6.64E-06 | -- | *ACOT4* | 5’ |
| rs7140346 | 14 | 74056648 | T | 159.97 (35.51) | 6.65E-06 | ++ | *ACOT4* | 5’ |
| rs10893764 | 11 | 127699872 | A | -108.67 (24.21) | 7.19E-06 | -- | - | - |
| rs141480969 | 12 | 62496278 | T | 372.96 (83.21) | 7.40E-06 | ++ | *FAM19A2* | intron |
| rs17713979 | 12 | 62501261 | T | -373.82 (83.44) | 7.46E-06 | -- | *FAM19A2* | intron |
| rs12478665 | 2 | 210123032 | T | 112.18 (25.08) | 7.72E-06 | ++ | - | - |
| rs28373101 | 14 | 74052588 | A | 168.51 (37.74) | 7.99E-06 | ++ | - | - |
| rs17714169 | 12 | 62513558 | A | -374.05 (83.77) | 8.00E-06 | -- | *FAM19A2* | intron |
| rs12148088 | 15 | 46209027 | T | -92.15 (20.65) | 8.07E-06 | -- | - | - |
| rs62495153 | 8 | 8136656 | T | -254.89 (57.2) | 8.35E-06 | -- | - | - |
| rs10851427 | 15 | 46208687 | A | 91.99 (20.65) | 8.38E-06 | ++ | - | - |
| rs80196851 | 2 | 173789113 | A | 460.86 (103.5) | 8.48E-06 | ++ | *RAPGEF4* | intron |
| rs10202661 | 2 | 210040339 | C | -93.23 (20.94) | 8.51E-06 | -- | *-* | *-* |
| rs13024041 | 2 | 205701987 | A | -121.26 (27.35) | 9.30E-06 | -- | *PARD3B* | intron |
| rs35567149 | 2 | 205764507 | A | 99.03 (22.35) | 9.42E-06 | ++ | *PARD3B* | intron |
| rs79613658 | 2 | 173885650 | A | 428.00 (96.67) | 9.54E-06 | ++ | *RAPGEF4* | intron |
| rs78073539 | 10 | 115378080 | A | -671.02 (151.73) | 9.75E-06 | -- | *NRAP* | intron |
| rs62179372 | 2 | 205764204 | A | -98.71 (22.34) | 9.90E-06 | -- | *PARD3B* | intron |
| rs13287452 | 9 | 11730087 | A | 471.97 (106.82) | 9.95E-06 | ++ | - | - |

**Notes.** SNP annotation information from SNPnexus [[1](#_ENREF_28)]; Effect direction is listed for the reported effect allele in MAS and OATs respectively

**References**

1. Dayem Ullah AZ, Lemoine NR, Chelala C (2013) A practical guide for the functional annotation of genetic variations using SNPnexus. *Brief Bioinform* **14**: 437-447.
