# Supplementary material for: Investigating the Genetics of Hippocampal Volume in Older Adults without Dementia
Source: PLoS One. 2015 Jan 27;10(1):e0116920. doi: 10.1371/journal.pone.0116920 (PMC4308067; doi:10.1371/journal.pone.0116920)
Supplement: S2 Table — (DOCX) [file pone.0116920.s002.docx]

**SUPPLEMENTARY MATERIAL**

**Table S2.** Cross-sectional hippocampal volume GWAS meta-analysis top results when using age, sex, scanner type and ICV as covariates

| **SNP** | **CHR** | **BP** | **Effect Allele** | **Beta (SE)** | ***p*-value** | **Effect Direction** | **Gene** | **Feature** |
| --- | --- | --- | --- | --- | --- | --- | --- | --- |
| rs8033195 | 15 | 94872686 | C | 173.86 (32.73) | 1.09E-07 | ++ | *MCTP2* | intron |
| rs59915516 | 15 | 94896171 | T | 163.75 (34.4) | 1.94E-06 | ++ | *MCTP2* | intron |
| rs17110850 | 1 | 94485491 | A | 131.94 (27.84) | 2.14E-06 | ++ | *ABCA4* | intron |
| rs13023239 | 2 | 2.06E+08 | A | -107.86 (22.77) | 2.17E-06 | -- | *PARD3B* | intron |
| rs13031723 | 2 | 2.06E+08 | T | 107.55 (22.87) | 2.56E-06 | ++ | *PARD3B* | intron |
| rs189456643 | 19 | 17872619 | A | -1061.57 (226.53) | 2.78E-06 | -- | *FCHO1* | intron |
| rs1019623 | 11 | 1.28E+08 | T | -98.56 (21.12) | 3.06E-06 | -- | - | - |
| rs72763355 | 1 | 2.39E+08 | A | -288.17 (61.77) | 3.09E-06 | -- | - | - |
| rs1378527 | 18 | 9709389 | C | -86.12 (18.8) | 4.61E-06 | -- | *RAB31* | intron |
| rs1863254 | 11 | 1.28E+08 | T | -96.36 (21.05) | 4.70E-06 | -- | - | - |
| rs28373101 | 14 | 74052588 | A | 163.16 (35.72) | 4.93E-06 | ++ | - | - |
| rs62078920 | 18 | 9708582 | T | -85.07 (18.63) | 4.96E-06 | -- | *RAB31* | intron |
| rs6054389 | 20 | 6592051 | A | 85.53 (18.75) | 5.09E-06 | ++ | - | - |
| rs77764430 | 9 | 122197445 | T | -324.73 (71.26) | 5.19E-06 | -- | - | - |
| rs1378528 | 18 | 9709481 | T | -84.18 (18.48) | 5.21E-06 | -- | *RAB31* | intron |
| rs62495153 | 8 | 8136656 | T | -246.61 (54.15) | 5.26E-06 | -- | - | - |
| rs71429703 | 2 | 2.07E+08 | A | -111.53 (24.49) | 5.29E-06 | -- | - | - |
| rs185977596 | 10 | 1.02E+08 | T | -1172.17 (257.73) | 5.42E-06 | -- | *DNMBP* | intron |
| rs992845 | 20 | 6607217 | A | -84.63 (18.61) | 5.44E-06 | -- | *-* | - |
| rs6117358 | 20 | 6592145 | A | 84.73 (18.64) | 5.49E-06 | ++ | *-* | - |
| rs13033719 | 2 | 2.06E+08 | A | -96.41 (21.22) | 5.51E-06 | -- | *PARD3B* | intron |
| rs6117359 | 20 | 6592422 | T | 84.70 (18.64) | 5.51E-06 | ++ | - | - |
| rs890134 | 11 | 1.28E+08 | T | 98.69 (21.73) | 5.56E-06 | ++ | - | - |
| rs994308 | 20 | 6603622 | T | 84.28 (18.57) | 5.64E-06 | ++ | - | - |
| rs4142038 | 20 | 6601732 | A | 84.17 (18.56) | 5.78E-06 | ++ | - | - |
| rs7361066 | 20 | 6586910 | T | 86.95 (19.21) | 6.03E-06 | ++ | - | - |
| rs144869364 | 11 | 30905802 | A | -515.32 (113.89) | 6.05E-06 | -- | *DCDC5* | intron |
| rs1863255 | 11 | 1.28E+08 | T | 100.28 (22.16) | 6.05E-06 | ++ | - | - |
| rs55951657 | 14 | 74074732 | A | -146.41 (32.37) | 6.11E-06 | -- | - | - |
| rs11221065 | 11 | 1.28E+08 | T | 96.90 (21.50) | 6.56E-06 | ++ | - | - |
| rs72930703 | 3 | 1E+08 | T | 717.70 (159.26) | 6.59E-06 | ++ | *TFG* | intron |
| rs10790922 | 11 | 1.28E+08 | A | -96.75 (21.47) | 6.63E-06 | -- | - | - |
| rs6054385 | 20 | 6587524 | T | -83.85 (18.61) | 6.64E-06 | -- | - | - |
| rs961676 | 20 | 6592621 | A | -83.43 (18.52) | 6.68E-06 | -- | - | - |
| rs6054399 | 20 | 6599698 | T | 83.55 (18.57) | 6.85E-06 | ++ | - | - |
| rs6117361 | 20 | 6606328 | A | 83.80 (18.65) | 6.98E-06 | ++ | - | - |
| rs2064857 | 20 | 6596582 | A | -82.97 (18.46) | 6.98E-06 | -- | - | - |
| rs1321454 | 20 | 6589099 | T | -83.57 (18.61) | 7.09E-06 | -- | - | - |
| rs2145278 | 20 | 6589373 | A | 83.57 (18.61) | 7.10E-06 | ++ | - | - |
| rs2326773 | 20 | 6590271 | A | 83.58 (18.61) | 7.10E-06 | ++ | - | - |
| rs2630772 | 12 | 54426521 | T | 102.13 (22.75) | 7.15E-06 | ++ | *HOXC6* | 3’ |
| rs61664876 | 8 | 3000240 | A | 112.48 (25.10) | 7.45E-06 | ++ | *CSMD1* | intron |
| rs2326771 | 20 | 6589726 | A | -83.55 (18.65) | 7.46E-06 | -- | - | - |
| rs7931878 | 11 | 1.28E+08 | T | -96.38 (21.55) | 7.75E-06 | -- | - | - |
| rs262147 | 7 | 1.59E+08 | T | 138.39 (30.96) | 7.82E-06 | ++ | - | - |
| rs7159970 | 14 | 74056288 | A | 150.30 (33.67) | 8.05E-06 | ++ | *-* | - |
| rs7140298 | 14 | 74056564 | T | 150.25 (33.66) | 8.05E-06 | ++ | *ACOT4* | 5’ |
| rs1893126 | 18 | 9712098 | T | 87.42 (19.59) | 8.08E-06 | ++ | *RAB31* | intron |
| rs11872195 | 18 | 9710887 | A | 87.35 (19.57) | 8.09E-06 | ++ | *RAB31* | intron |
| rs1455587 | 18 | 9710554 | T | 87.31 (19.56) | 8.09E-06 | ++ | *RAB31* | intron |
| rs28494539 | 14 | 74054934 | T | -150.44 (33.71) | 8.11E-06 | -- | *-* | - |
| rs12588475 | 14 | 74056820 | A | 149.74 (33.59) | 8.27E-06 | ++ | *ACOT4* | 5’ |
| rs7160971 | 14 | 74056604 | A | -149.82 (33.61) | 8.30E-06 | -- | *ACOT4* | 5’ |
| rs7140346 | 14 | 74056648 | T | 149.77 (33.6) | 8.31E-06 | ++ | *ACOT4* | 5’ |
| rs8018361 | 14 | 74073502 | T | -144.02 (32.33) | 8.42E-06 | -- | - | - |
| rs2009884 | 14 | 74061490 | T | -145.35 (32.65) | 8.50E-06 | -- | *ACOT4* | intron |
| rs8019162 | 14 | 74073324 | T | 143.85 (32.32) | 8.55E-06 | ++ | - | - |
| rs6574129 | 14 | 74066812 | T | -143.85 (32.32) | 8.55E-06 | -- | - | - |
| rs9671218 | 14 | 74065505 | A | -143.88 (32.33) | 8.60E-06 | -- | - | - |
| rs7931009 | 11 | 1.28E+08 | T | -95.78 (21.53) | 8.66E-06 | -- | - | - |
| rs80234352 | 20 | 6592187 | A | 83.84 (18.85) | 8.72E-06 | ++ | - | - |
| rs28841877 | 3 | 1.28E+08 | A | 109.70 (24.68) | 8.81E-06 | ++ | - | - |
| rs12951929 | 17 | 65774409 | C | -107.17 (24.12) | 8.85E-06 | -- | - | - |
| rs12101151 | 14 | 74064151 | T | -144.54 (32.53) | 8.87E-06 | -- | *ACOT4* | 3’ |
| rs55689428 | 14 | 74064245 | T | -144.48 (32.52) | 8.88E-06 | -- | *ACOT4* | 3’ |
| rs12226793 | 11 | 1.28E+08 | A | -96.25 (21.67) | 8.90E-06 | -- | - | - |
| rs11079703 | 17 | 65797833 | T | -99.44 (22.39) | 8.93E-06 | -- | - | - |
| rs11651507 | 17 | 65801030 | C | 99.27 (22.36) | 9.02E-06 | ++ | - | - |
| rs9672127 | 14 | 74065221 | A | -144.10 (32.46) | 9.03E-06 | -- | - | - |
| rs6504535 | 17 | 65773808 | T | 106.71 (24.05) | 9.09E-06 | ++ | - | - |
| rs12991740 | 2 | 1.73E+08 | T | 84.59 (19.06) | 9.09E-06 | ++ | *METAP1D* | intron |
| rs857160 | 1 | 57241867 | T | -91.58 (20.67) | 9.37E-06 | -- | *C1orf168* | intron |
| rs12587542 | 14 | 74067562 | A | -143.24 (32.33) | 9.40E-06 | -- | - | - |
| rs141743738 | 8 | 11023948 | T | -762.88 (172.25) | 9.47E-06 | -- | *XKR6* | intron |
| rs1774817 | 1 | 57243086 | T | -91.40 (20.67) | 9.75E-06 | -- | *C1orf168* | intron |
| rs2365412 | 17 | 65794209 | A | 99.63 (22.54) | 9.91E-06 | ++ | - | - |

**Notes.** SNP annotation information from SNPnexus [[1](#_ENREF_28)]; Effect direction is listed for the reported effect allele in MAS and OATs respectively.

**References**

1. Dayem Ullah AZ, Lemoine NR, Chelala C (2013) A practical guide for the functional annotation of genetic variations using SNPnexus. *Brief Bioinform* **14**: 437-447.
