# Supplementary material for: Investigating the Genetics of Hippocampal Volume in Older Adults without Dementia
Source: PLoS One. 2015 Jan 27;10(1):e0116920. doi: 10.1371/journal.pone.0116920 (PMC4308067; doi:10.1371/journal.pone.0116920)
Supplement: S3 Table — (DOCX) [file pone.0116920.s003.docx]

**SUPPLEMENTARY MATERIAL**

**Table S3.**  Top GWAS results for hippocampal atrophy over two years for Sydney MAS participants with age and sex as covariates

| **SNP** | **CHR** | **BP** | **P-value** | **Alleles** | **Gene** | **Feature** |
| --- | --- | --- | --- | --- | --- | --- |
| rs11799420 | 1 | 171652978 | 4.07E-06 | C/T | - | - |
| rs11580415 | 1 | 171666832 | 4.07E-06 | A/G | - | - |
| rs11579698 | 1 | 171666922 | 4.07E-06 | C/T | - | - |
| rs9662853 | 1 | 171652731 | 4.07E-06 | A/G | - | - |
| rs6667559 | 1 | 171653957 | 4.42E-06 | C/T | - | - |
| rs11590714 | 1 | 171716779 | 4.51E-06 | C/G | - | - |
| rs41349744 | 11 | 74052264 | 5.03E-06 | C/G | *PGM2L1* | intron |
| rs10913582 | 1 | 171715585 | 5.82E-06 | A/G | - | - |
| rs10913511 | 1 | 171668951 | 7.03E-06 | A/G | *VAMP4* | 3’ |
| rs10913529 | 1 | 171679465 | 7.03E-06 | A/C | *VAMP4* | intron |
| rs17649050 | 1 | 171713808 | 7.03E-06 | C/G | - | - |
| rs11577073 | 1 | 171716724 | 7.03E-06 | C/T | - | - |
| rs17649250 | 1 | 171720586 | 7.03E-06 | C/G | - | - |
| rs11577745 | 1 | 171720909 | 7.03E-06 | C/G | - | - |
| rs6679027 | 1 | 171733094 | 7.86E-06 | G/T | - | - |
| rs3897548 | 11 | 73968836 | 8.90E-06 | A/G | *P4HA3* | intron |
| rs1531462 | 8 | 76752790 | 9.07E-06 | A/G | - | - |
| rs11242023 | 5 | 129661732 | 9.55E-06 | C/T | - | - |
| rs9589896 | 13 | 94731597 | 9.81E-06 | C/T | *GPC6* | intron |

**Notes**. SNP annotation information from SNPnexus [[1](#_ENREF_28)]

**References**

1. Dayem Ullah AZ, Lemoine NR, Chelala C (2013) A practical guide for the functional annotation of genetic variations using SNPnexus. *Brief Bioinform* **14**: 437-447.
