# Supplementary material for: Investigating the Genetics of Hippocampal Volume in Older Adults without Dementia
Source: PLoS One. 2015 Jan 27;10(1):e0116920. doi: 10.1371/journal.pone.0116920 (PMC4308067; doi:10.1371/journal.pone.0116920)
Supplement: S4 Table — (DOCX) [file pone.0116920.s004.docx]

**SUPPLEMENTARY MATERIAL**

**Table S4.**  Top GWAS results for hippocampal atrophy over two years for Sydney MAS participants with age, sex and ICV as covariates

| **SNP** | **CHR** | **BP** | **P-value** | **alleles** | **gene** | **feature** |
| --- | --- | --- | --- | --- | --- | --- |
| rs1531462 | 8 | 76752790 | 2.73E-06 | A/G | - | - |
| rs41349744 | 11 | 74052264 | 5.74E-06 | C/G | *PGM2L1* | intron |
| rs11242023 | 5 | 129661732 | 6.71E-06 | C/T | - | - |
| rs11579698 | 1 | 171666922 | 6.82E-06 | C/T | - | - |
| rs11580415 | 1 | 171666832 | 6.82E-06 | A/G | - | - |
| rs11799420 | 1 | 171652978 | 6.82E-06 | C/T | - | - |
| rs9662853 | 1 | 171652731 | 6.82E-06 | A/G | - | - |
| rs9589896 | 13 | 94731597 | 7.28E-06 | C/T | *GPC6* | intron |
| rs6667559 | 1 | 171653957 | 7.35E-06 | C/T | - | - |
| rs11590714 | 1 | 171716779 | 7.58E-06 | C/G | - | - |
| rs3897548 | 11 | 73968836 | 8.78E-06 | A/G | *P4HA3* | intron |
| rs10054055 | 5 | 129651722 | 8.92E-06 | C/T | - | - |
| rs10913582 | 1 | 171715585 | 9.36E-06 | A/G | - | - |
| rs1794681 | 6 | 33579661 | 9.37E-06 | C/T | - | - |
| rs6679027 | 1 | 171733094 | 9.78E-06 | G/T | - | - |
| rs12153185 | 5 | 129666393 | 1.03E-05 | C/T | - | - |

**Notes.** ICV = intracranial volume; SNP annotation information from SNPnexus [1]

**References**

1. Dayem Ullah AZ, Lemoine NR, Chelala C (2013) A practical guide for the functional annotation of genetic variations using SNPnexus. *Brief Bioinform* **14**: 437-447.
