# Supplementary material for: Investigating the Genetics of Hippocampal Volume in Older Adults without Dementia
Source: PLoS One. 2015 Jan 27;10(1):e0116920. doi: 10.1371/journal.pone.0116920 (PMC4308067; doi:10.1371/journal.pone.0116920)
Supplement: S2 Fig — P-values for each individual SNP are plotted against chromosome position from the results of the association analysis with bilateral hippocampal volume. The analysis was adjusted for age, sex, scanner and ICV. (DOCX) [file pone.0116920.s006.docx]

**SUPPLEMENTARY MATERIAL**


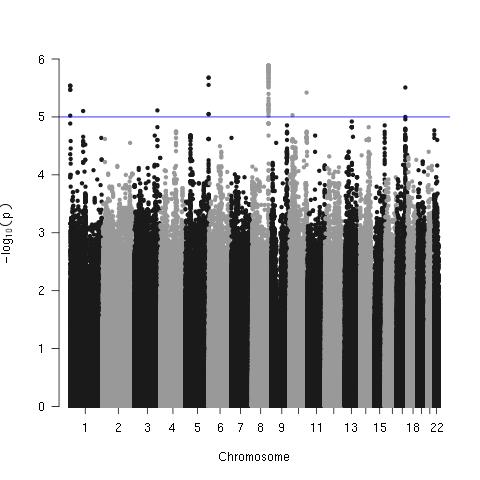


Figure S2. Genome-wide Manhattan plot for hippocampal volume using the Older Australian Twins Cohort. *P*-values for each individual SNP are plotted against chromosome position from the results of the association analysis with bilateral hippocampal volume. The analysis was adjusted for age, sex, scanner and ICV.
